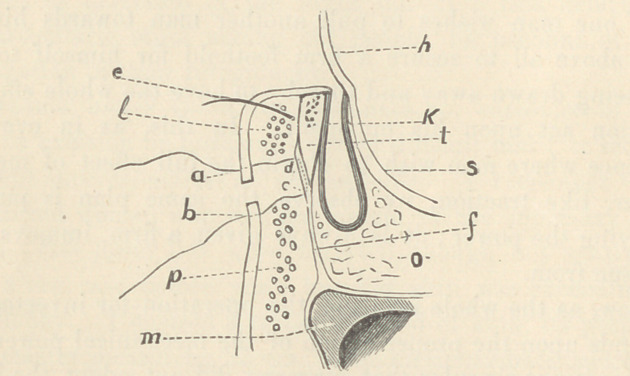# A Clinical Lecture upon the Operation for Inversion of the Lower Eyelid

**Published:** 1880-01

**Authors:** F. C. Hotz

**Affiliations:** Delivered at the Illinois Charitable Eye and Ear Infirmary


### CHICAGO MEDICAL JOURNAL & EXAMINER.

VOL. XL.—JANUARY, 1880.—No. 1.

[ n these 'pages dimension and weight are expressed in terms of the Metric System, and temperature in degrees of the Centigrade Scale.]

### Original Lectures.

### ARTICLE I.

A CLINICAL LECTURE UPON THE OPERATION FOR INVERSION OF THE LOWER EYELID. By F. C. HOTZ, M.D. Delivered at the Illinois Charitable Eye and Ear Infirmary, November 13, 1879.

GENTLEMEN: In comparing the right and left eye of this patient, you will notice a remarkable difference in the appearance of the lower eyelids. While the left lower eyelid appears normal in every way, the right one seems to be reduced to an unsightly thick piece of integument. You cannot dectect any eyelashes, you cannot see anything like the free edge of the lid. It looks as if the whole lid had been destroyed, and the external integument were drawn inward by the cicatrization and directly united with the conjunctiva. But now let me press slightly upon this rounded border of the skin, and you will perceive a great transformation; as if I had touched a secret spring, the lid border together with the eyelashes suddenly turn up from behind the skin; and now the right lower eyelid looks just like its partner, with the cilia well developed and grown in a regular row from a smooth, clean-cut edge. If I discontinue the pressure upon the skin, the lid border with its eyelashes slowly sinks down again, to disappear behind the skin which rolls in after it.

We have here a typical case of a complete inversion of the lower lid. It is rolled in upon itself in such manner that its free edge and eyelashes are turned down toward, and buried in, the lower cul-de-sac of the conjunctiva. The patient informed me that he had been afflicted with granular conjunctivitis for twenty years, and that the right lower lid had been inverted for at least six or seven years. I mention these data, because the chronicity of the affection, in connection with the readiness with which the lid turns in again after it has been everted, and the absence of spasms of the orbicular muscle—these facts must lead us to suppose that organic changes have taken place in the tissues which compose this lower lid, of such a character that the inversion is a permanent condition. It is, therefore, beyond the influence of medicinal application; nor would it be materially affected by our curing of the granular conjunctivitis, if such a thing were possible under the circumstances. But as long as the inverted eyelashes continue their mechanical irritation of the conjunctiva, so long any treatment of the conjunctival disease will meet with little or no success. The first step, therefore, in dealing with a case like this, is to remedy the entropion, because thereby we remove one of the greatest obstacles to a successful treatment of the granular conjunctivitis.

The common practice in cases of chronic, confirmed inversion of the lower eyelid, which require operative treatment, is either to excise one transverse fold or several longitudinal folds of the integument of the lid, or to insert through the basis of a transverse fold two or three ligatures. The wounds created by the removal of skin are allowed to heal by granulation; and the ligatures, being drawn as tightly as possible, remain in place until they are eliminated by suppuration. Thus, either by scissors or by ligatures, wounds are inflicted upon the skin of the lower lid, which are repaired by granulation tissue. Cicatricial tissue, resulting from such wounds, always shrinks a good deal, drawing with an irresistible force the surrounding tissues toward the center of the scar, or, if one end of the scar is attached to some immovable substance, toward this fixed point. In the case of the lower lid, the scars established by the operations mentioned are connected, above with the free edge of the lid, and below with the integument of the cheek. By the cicatricial contraction, therefore, these two parts are drawn toward each other, and, if the integument of the cheek be firm and comparatively unyielding, the lid border, on account of its great mobility, becomes principally affected by the cicatricial tension. From its normal position it would be drawn away so as to become everted; but if inverted it may perhaps be turned just enough to recover its normal position.

You will have perceived, gentlemen, that the theory underlying all these operations, is to employ the contracture of cicatricial tissues as the power with which the reposition of the inverted eyelid shall be accomplished. This end, however, is quite often not attained, because, as I think, there are two very serious defects in the plan upon which these operations are executed.

1. *The effect of the operation depends upon the contraction of cicatricial tissues.* That is to say, we have to rely upon one unknown quantity, an x, as mathematicians call it. For we cannot know how much or how little the scar of a granulating wound of a certain length or width may shrink; we have no influence upon this shrinkage: we can neither increase nor arrest it. We entrust, therefore, the ultimate result of our operation to an agency which is entirely beyond our control; and consequently we must not be surprised at all if our calculation, based upon one x, shows up a different result from that which we bargained for.

2. *The result of these operations is too dependent upon the state of the integument of the cheek.* This is a worse fault than the other, because it makes, in most cases, a permanent success illusory. Let us review the mechanical effect of the operation: By excision or ligation, the skin of the lower lid is shortened in the longitudinal direction for the purpose of making the requisite traction upon the edge of the inverted eyelid to turn it back to its proper position. But, in order to make this traction effectively and successfully, the opposite end of the contracting cicatricial band must be firmly and immovably fixed; if it is not, the traction will operate upon both ends, and the greater degree of mobility alone will decide which will be moved the most. If the integument of the cheek, to which the lower end of the cicatricial band is attached, is solid and firm, it forms a pretty good basis for the traction. But let that integument be very flaccid, as we find it in aged people; let at the same time the lid be held inverted by extensive atrophic shrinkage of the conjunctiva, and the traction of the cicatricial band will draw the flaccid and movable skin of the cheek upward rather than the lid border downward. And this is just the condition which obtains in many cases of inversion of the lower lid. For this deformity occurs particularly often among old people, on account of the great relaxation of their integument.

To overcome, under these circumstances, an entropion of the lower lid by excision or ligation is a very difficult task; to secure a permanent success, almost an impossibility. I remember very well yet an old woman who was an inmate of the infirmary two years ago. Her lower lids were inverted, though not nearly so badly as in the case before you; but her skin was exceedingly thin and flabby. I excised a transverse fold of skin, its perpendicular diameter measuring 2½ cm., with an immediate success; but two months later the lids were inverted again. I removed another piece of skin of equal dimensions; and in the course of a few months the effect of the second operation also was reduced to a mere shadow. The enormous shortening of the skin had no permanent effect upon the lid border because the relaxed skin evidently was more easily moved by the shrinking scars than the edge of the lid.*

I admit the instance just related belonged to the bad cases of

* At the following clinique, Dr. Hotz exhibited a case of recurrent inversion of the lower lid to substantiate the above remarks. The patient, 60 years old, had granular conjunctivitis and a marked degree of entropion of both lower lids; the skin of his cheeks was very sallow, exceedingly flaccid and wrinkled. Five or six millimeters below the free edge of each lower lid there was a callous transverse scar running across the whole width of the lid. This scar was the result of an excision of skin for the cure of the entropion, performed five years ago. So lax was the skin that if a piece of skin, 2 cm. in vertical diameter, was taken up in a transverse fold, this shortening barely sufficed to evert the lid; while it was easily done without any sacrifice of integument by the operation described below.

entropion, and that in less difficult cases the same operation has often been performed with unquestionable success. But to me bad cases are always the most interesting because the most instructive. They are the only cases by which the merits of an operation can be fairly tested, because if there be any fault in the conception or execution of an operative method, they will bring it out in strong relief. If an operation cures mild degrees of inversion of the lower lid, but fails with more pronounced cases, I come to the conclusion that there is something wrong in the principle of that operation. And I believe it is not difficult to show that the frequent failures of the operations for entropion are undoubtedly due to the wrong or disadvantageous application of the principle of traction.

If one man wishes to pull another man towards himself, he tries above all to secure a firm foothold for himself to prevent his being drawn away and thereby to have the whole effort of his traction act upon his opponent. In this, as in every other instance where men wish to obtain the full effect of mechanical power, like traction, we observe the same plan is pursued in applying the power; it is always given a firm, immovable basis to issue from.

Now, as the whole result of the operation for inverted eyelids depends upon the proper action of the mechanical power of traction, is it not singular that surgeons did not adopt the best way of using mechanical power with the greatest advantage? Is it not strange that instead of securing a firm, anyielding basis for the lower end of the cicatrix, they committed the great blunder of affixing both ends of the contracting cicatrix to mobile structures? The natural and necessary consequence was that the traction worked both ways; its impulse was perceptible upon the integument of the cheek as well as upon the edge of the lid and the greater mobility of either alone determined which would be displaced the most.

This is a very unsatisfactory way of doing things. The effect of an operation upon so important an organ as the eye, should not be left to the influence of casual circumstances over which we have no control. If we employ the mechanical principle of traction for the cure of inversion of the lower lid, let us apply it in such a way that we can determine its effect with an almost mathematical accuracy. *This is possible only if we obtain a fixed immovable point toward which the skin of the lid can be drawn and to which it can be so fastened that a permanent traction is made upon the free border of the lid.*

*Such a point is the lower border of the tarsal cartilage.*

To recall to your mind the necessary anatomical data, let me say that the lower lid is composed of the following layers: external integument; subcutaneous cellular tissue; orbicular muscle; another layer of cellular tissue; tarsal cartilage or tarsus; and conjunctiva. To the lower border of the tarsus ($t$) is attached a strong fascia ($f$), the fascia tarso-orbitalis, which, extending from the tarsal cartilage to the infra-orbital margin ($m$) of the maxillary bone closes the lower part of the orbital cavity ($o$) against the pars orbitalis ($p$) of the orbicular muscle. At the osseous margin this fascia passes over into the periosteum; at the tarsus it is blended with its lower border and also spread upon the lower third of its anterior surface. Owing to this fascial connection, the lower border of the tarsus can firmly resist any power which tends to move it upward, because through the fascia this upward movement is impeded by the infra-orbital margin. For this reason the lower border of the tarsus itself is an immovable point best suited as a basis for the traction which is to stretch the skin of the

---

The above diagram represents a vertical section of the lower lid and its surroundings; *k*, conjunctiva of lid; *t*, tarsal cartilage; *e*, eyelash; *l*, lid portion of the orbicular muscle; *p*, pars orbitalis of same muscle; *f*, fascia tarso-orbitalis; *c*, *d*, expansion of fascia upon lower third of tarsus; *m*, infra-orbital margin; *o*, orbital tissue; *s*, sclerotic; *h*, cornea; *a d c b*, course of the suture.

lower eyelid and to draw its free edge downward and outward in case of inversion.

This firm basis gained, a very slight traction suffices for the reposition of the inverted eyelid. I will demonstrate it on the patient before you. The lower border of the tarsus of the lower lid describes a slightly curved line with its convexity looking downwards; its place is indicated by a fine, slightly curved furrow more or less well marked in the skin, and from 4 to 6 m. m. below the edge of the lid. Now, I put this silver probe in the furrow and with the slightest possible pressure I push the skin directly downwards and backwards. The movement of the skin thus produced is scarcely perceptible, and yet it is sufficient to turn the lid border. It has now a normal position.

That you may fully realize the importance of this experiment, let me show you, by way of contrast, what a large piece of skin would have to be removed to obtain the same result. I must take up a transverse fold of as large a piece of integument as the open prongs of these forceps can grasp, in order to turn the inverted lid border. All this skin would have to be excised, were we to follow the common practice in the treatment of this case. But over one year ago I came to the conclusion that the excision of skin was an unnecessary mutilation ; that our object could be accomplished with greater nicety and certainty without shortening the skin. I have since departed from the high road of common practice, and the larger my experience grew, the better I was pleased with the new method of operating for inversion of the lids. In the *Archives of Ophthalmology*, Vol. viii, No. 2, you will find a description of this operation for the entropion of the upper lid. And I shall presently show you how the same operation is performed upon the lower lid.

Four or six millimeters below the lid border (just along that fine furrow I referred to before), I make a transverse incision, which severs the skin of the lid from the integument of the cheek. An assistant then draws with a forceps the upper border of the wound upwards, while I am drawing the lower border downwards. Thus the wound is widely opened and the orbicular muscle fully exposed. A few horizontal strokes of the scalpel near the upper border of the wound open the muscular layer and submuscular cellular tissue and expose the lower border of the tarsus to its whole extent. The tarsus is easily recognized by its yellowish color; but if you are in doubt, just feel with your finger to detect the firm edge which differs quite perceptibly from the soft elastic resistance of the fascia below.

The bundles of muscular fibers which covered the lower third of the tarsus, must now be removed so that the skin and tarsus can be brought in direct apposition. As a rule, I find, after the incision of the orbicularis, no muscular fibers upon the surface of the tarsus; but find them attached to, and drawn up with, the tarsal skin. With a pair of fine forceps I seize the muscular fibers near the upper border (*a*) of the skin and excise a strip, about 3 m. m. in width, along the whole length of the lid from canthus to canthus. This done, the wound is ready for the final and most important step of the operation, the application of the sutures. The curved needle armed with fine black silk * is thrust through the skin of the lid, a few m. m. from the border of the incision (at *a*); then it pierces the fascial expansion upon the lower third of the tarsus (at *d*) and is carried downward along the tarsus until its point emerges again (at *c*) from the fascia a little below the tarsal border; and finally it is passed through the lower border of the wound (at *b*), great care being taken to exclude all muscular fibers from the loop of the thread. The suture (*a*, *d*, *c*, *b*,) thus inserted, includes only the two cutaneous borders and a piece (*d c*) of the fascia, and when it is tied, it brings the cutaneous borders together and also draws them down to the tarsus.

Three sutures of this kind usually are sufficient for uniting the skin with the whole length of the tarsal border. During the first twenty-four hours after the operation the eye-lids are kept covered with cold water compresses; they relieve the smarting sensation and probably also mitigate the inflammatory reaction. On the second day the lids are found slightly swollen and tender; this swelling and tenderness are a little greater on the third day, but subside quickly after the removal of the stitches. As a rule the sutures are taken out on the third day.

---

*I use black thread for the great advantage it has over light colored silk, in the easy removal of the sutures.

And now, while our patient is being etherized, I invite you to examine a few eye-lids upon which this operation has been performed sometime ago. In none of these cases has the skin been mutilated by excision or ligation; in every one the operation has been successful. And I wish to turn your attention particularly to the firm union which is established between the skin and the lower border of the tarsus at the line of the operation, marked out by this linear transverse scar. The firmness of this union and the important rôle that it plays, is manifested when you push the integument of the cheek upwards. You may do so as much as you please, and you notice this has no influence upon the position of the lid border. It does not turn in, no matter how much you relax the tissues of the cheek, which it would do very readily, if its eversion had been brought about by the contraction of a cutaneous cicatrix.

In the beginning of this lecture I mentioned, as the greatest defect of the old operations, that their result was too dependent upon the state of the integument of the cheek, and that in this dependence I recognize a fruitful source of the recurrence of entropion. This new method has made the result of the operation wholly independent of the condition of the surrounding tissues; and this independence is a sure guarantee that the pains of the surgeon are not expended for an ephemeral result, but that his labor will be rewarded by a permanent success.

---

THE students of the Woman's Medical College and of the Rush Medical College had the pleasure of witnessing, on Monday evening, December 22, an exhibition of a beautiful series of micro-photographs prepared and kindly loaned by Dr. Walter Kempster, the efficient Superintendent of the Hospital for the Insane near Oshkosh, Wisconsin. These excellent pictures were prepared by Dr. Kempster to illustrate his article published in the proceedings of the International Medical Association in 1876. They demonstrate conclusively that the doctor's capacity extends beyond the duties of a medical superintendent, and place him in the front rank of American histologists.

## Figures and Tables

**Figure f1:**